# Supplementary material for: Full hardware implementation of neuromorphic visual system based on multimodal optoelectronic resistive memory arrays for versatile image processing
Source: Nat Commun. 2023 Dec 20;14:8489. doi: 10.1038/s41467-023-43944-2 (PMC10733375; doi:10.1038/s41467-023-43944-2)
Supplement: Supplementary file 1 — Supplementary Information [file 41467_2023_43944_MOESM1_ESM.pdf]

Supplementary Information

Full Hardware Implementation of Neuromorphic Visual System Based on Multimodal Optoelectronic Resistive Memory Arrays for Versatile Image Processing

Guangdong Zhou,[1] Jie Li,[2] Qunliang Song,[3] Lidan Wang,[1] Zhijun Ren,[1] Bai Sun,[4] Xiaofang Hu, [1] Wenhua Wang,[3] Gaobo Xu,[3] Xiaodie Chen,[5] Lan Cheng,[6] Feichi Zhou[2]* & Shukai Duan[1]*

[1] College of Artificial Intelligence, Key Laboratory of Brain-like Computing and Intelligent Control (Chongqing), Key Laboratory of Luminescence Analysis and Molecular Sensors (Ministry of Education), Southwest University, Chongqing, 400715, China.

[2] School of Microelectronics, Southern University of Science and Technology, Shenzhen, 518055, China.

[3] Faculty of Materials and Energy, Southwest University, Chongqing, 400715, China.

[4] Frontier Institute of Science and Technology, Xi'an Jiaotong University, Shanxi 710049, China.

[5] Department of Electrical and Electronic Engineering, The University of Hong Kong, Hong Kong, China.

[6] State Key Laboratory of Silkworm Genome, College of Sericulture, Textile and Biomass Sciences, Southwest University, Chongqing, 400715, China.

*e-mail: duansk@swu.edu.cn; zhoufc@sustech.edu.cn

Supplementary Note 1

MSFP synthesis and characterizations. Supplementary Figure 1a shows an optical image of the Au/MSFP/Au memory crossbar array with a scale of 12×12, showing an overall footprint of 4.6mm×4.6mm. The area of a single memory cell is 200μm ×200μm, as shown in Supplementary Figure 1b.

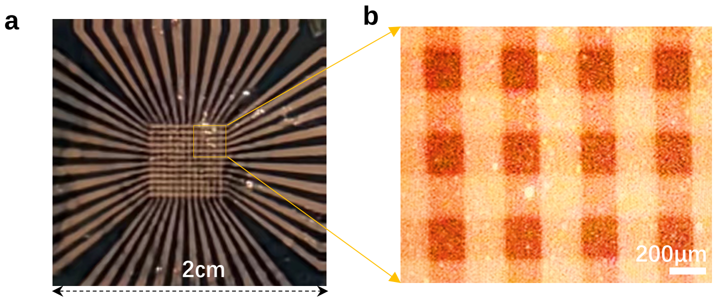

Supplementary Fig. 1. a. Optical image of a 12× 12 Au/MSFP/Au resistive memory crossbar array. **b**. Optical microscopy image of the Au/MSFP/Au memory cells with an effective area of 200 μm ×200 μm in a single cell.

Supplementary Figure 2 shows the synthesis of the modified silk fibroin protein (MSFP) thin film and flexible MSFP substrate. The silk can be obtained from the cocoon of the practical silkworm strain LiangGuang-No.2 (LG-2) (Supplementary Figure 2a). After the standard processing[1-4], the silk fibroin protein (SFP) solution was obtained (left of Supplementary Figure 2b). After adding the Pg-3 and 5, 6-DHI into the SFP solution (including 2mL deionized water) according to the mass ratio of 0.0025, 0.0025 and 0.028g, the black MSFP precursor solution was obtained (right of Supplementary Figure 2b). The MSFP switching thin film can be obtained after spin-coating, while the flexible MSFP substrate was synthesized after thermal processing at 97°C for 3 hours (Supplementary Figure 2c). The fabricated MSFP substrate exhibits

good flexibility (Supplementary Figures 2d-f), which can be ascribed to the rich hydrogen bonds and hydroxyls that are provided by the Pg-3 and 5, 6-DHI. The MSFP thin film synthesized by the SFP solution, Pg-3 and 5, 6-DHI, showed a uniformly smooth and dense surface (Supplementary Figures 2g-i).

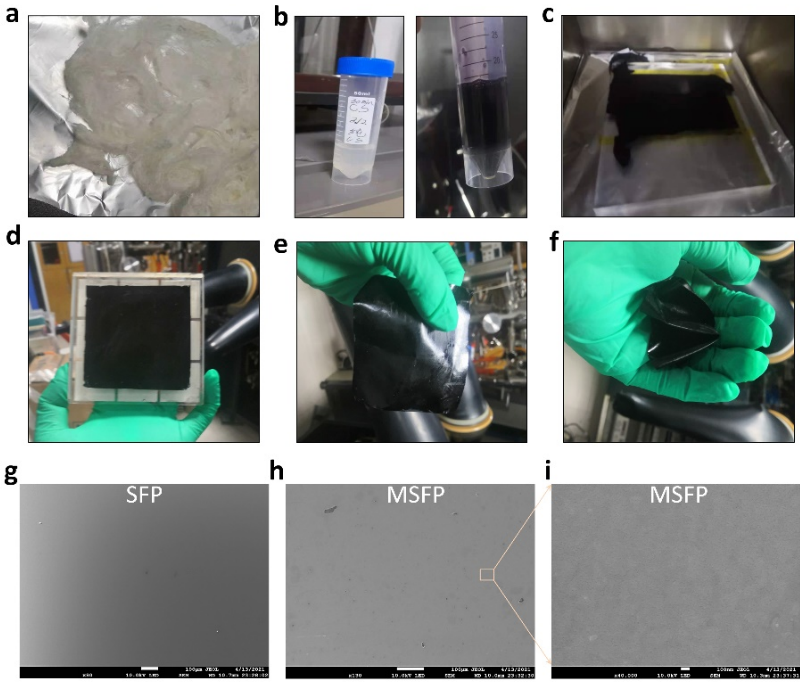

Supplementary Fig. 2. Synthesis of MSFP switching thin films. a. Natural silk from the cocoon LG-2. **b**. Fibroin extracted from cocoon LG-2 and precursor solution of the MSFP. **c**. Preparation of the flexible MSFP substrate after thermal processing. **d-f**. Optic images of the synthesized MSFP substrate. **g-i**. FE-SEM images of the MSFP film spin-coated by the MSFP precursor solution. The film presents a dense and smooth state.

The XRD and SEM characterized the changes in crystallographic and structural properties of SFP and MSFP thin films. Supplementary Figure 3a exhibits the XRD patterns of the SFP thin film prepared under different conditions, including the precursor dialyzing time of 12, 24, 36, 48 and 72 hours, a mixture of Pg-3 and SFP with the dialyzing time of 48 hours, a mixture of 5,6-DHI and SFP the dialyzing time of 48

hours, and the MSFP thin film with the SFP precursor dialyzing of 48 hours. No noticeable crystallographic changes are detected between SFP and MSFP thin films. Supplementary Figures 3b-f show the field emission scanning electron microscope (FE-SEM) images of the SFP sample with different precursor dialyzing times from 12 to 72 hours, showing no obvious change in the SFP surface. After reaction with the Pg-3, the 5, 6-DHI, or both of them, the formed MSFP film surface becomes denser and smoother, showing higher thin film quality (Supplementary Figures 3g-i), which may be responsible for the lower current density and higher stability in the MSFP memory device compared with that in the SFP memory device.

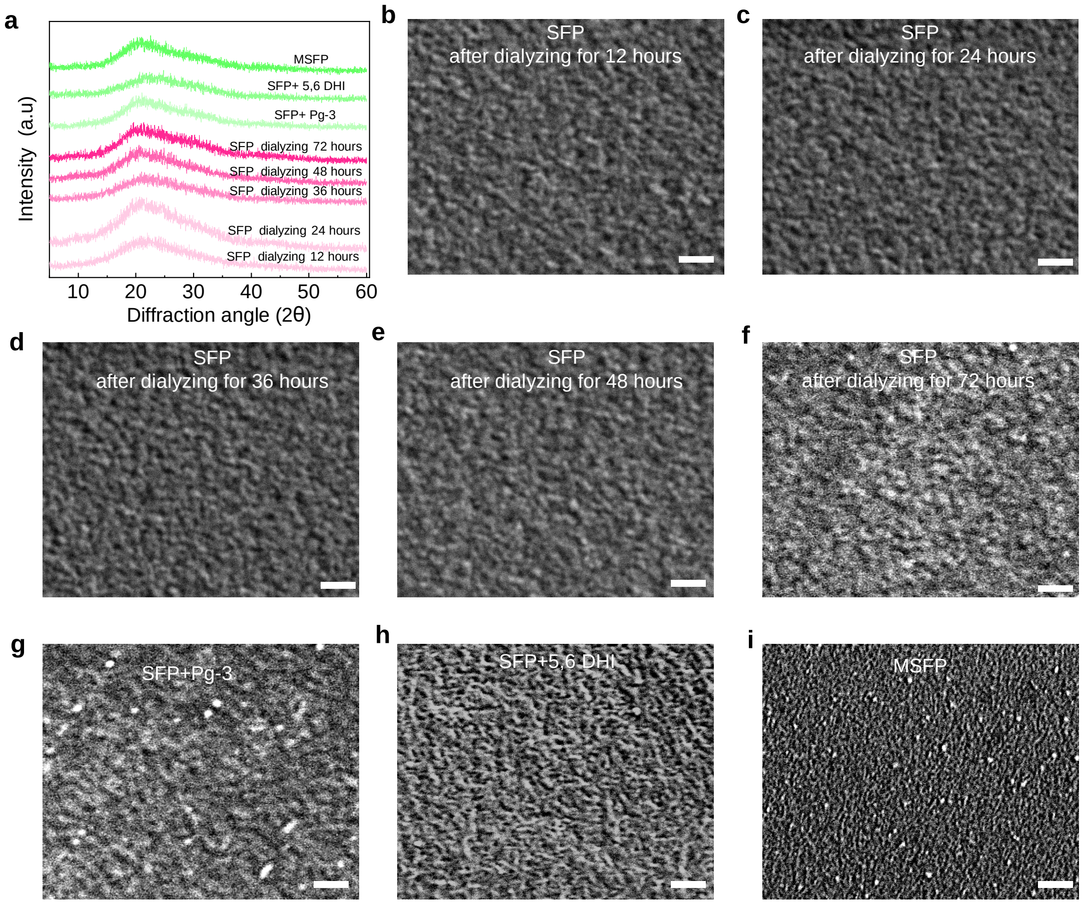

Supplementary Fig. 3. Crystallographic and structural characterizations of SFP and MSFP thin films. a. The XRD pattern of the SFP thin film with various precursor dialyzing times from 12 to 72 hours and the MSFP thin film formed by the reaction of Pg-3, 5,6 DHI with SFP. **b-f**. FE-SEM images

of the USFP thin films with dialyzing times of 12, 24, 36, 48, and 72 hours, respectively. **g-j**. FE-SEM image of the SFP thin films and MSFP thin film (dialyzing time of 48 hours), respectively. The scale bar represents 500 nm.

The FTIR results between SFP and MSFP demonstrated that the strong chemical bond vibration of the -OH, -C=O, C=C, and C-OH respectively located at 3278, 1635, 1520 and 1230 cm^{-1} are observed in both SFP and MSFP thin films while a weak -CH_2(s) vibration located at 2856 cm^{-1} is observed in the MSFP thin film, indicating that the major structures of the SFP can be reserved in the MSFP after the chemical reaction between SFP, 5,6-DHI and Pg-3 and the structures such as the connection between protein chains in the MSFP are changed during the reaction. In addition, the chemical bond of C-O located at 1165 cm^{-1} in the SFP is not detected in the MSFP (Supplementary Figure 4), indicating that the C-O-based bond groups can possibly provide the chemical reaction sites to form a large number of hydrogen bonds among USFP, Pg-3 and 5,6-DHI [5-8].

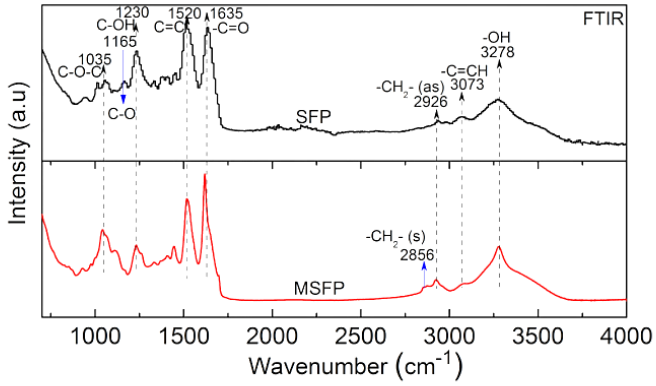

Supplementary Fig. 4. FTIR spectra of the SFP and MSFP. The primary structures of SFP were reserved in the MSFP thin film; however, the –CH_2-based chains were generated in the MSFP thin film.

The MSFP thin film shows good dielectric properties and strong absorption in the visible light region. The SFP material's dielectric constant (εr) is 8.88 @ 1MHz, consistent with the previous reports [6]. The synthesized MSFP material shows a higher dielectric constant of 40.62@ 1MHz, which exceeds four times than the SFP thin film (Supplementary Figure 5a), enabling the MSFP-based electronic device with good electrical properties such as bias durability, low leakage current, extensibility, and higher charge binding capability [3]. The absorption spectra of MSFP and SFP thin films on the quartz substrate are shown in Supplementary Figure 5b. The MSFP material exhibits stronger absorption in the visible light range than the SFP thin film, enabling the MSFP-based resistive memory with good optoelectronic properties.

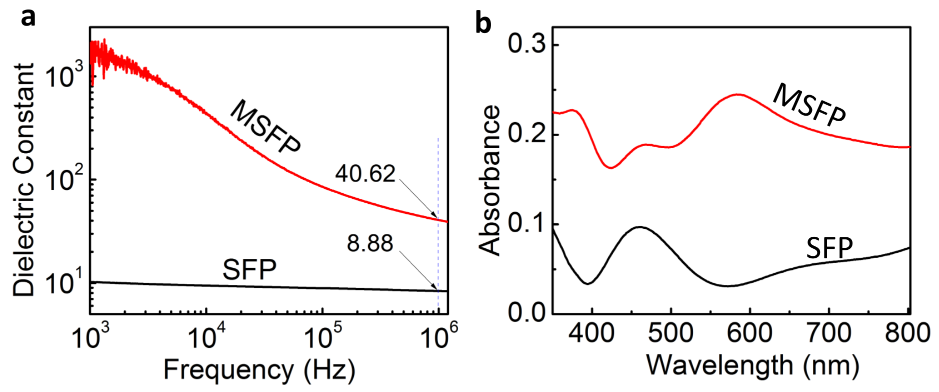

Supplementary Fig. 5. Dielectric properties and light absorption spectra of the SFP and MSFP thin films. a. Dielectric constant *versus* frequency for the SFP and MSFP thin films. The permittivity of 40.62 was obtained @ 1MHz for the MSFP thin film, which exceeds four times that in the SFP. **b**. Absorption spectra of SFP and MSFP thin films in the visible region.

Supplementary Note 2

Electrical resistive switching. Supplementary Figure 6a shows the cyclic test of the electrical switching for 1000 cycles, indicating good endurance. Supplementary Figure

6b shows the HRS and LRS retention properties programmed by voltage sweepings in Figure 2a. The cumulative probability of the LRS and HRS for the 100 memristor cells under electric stimuli are operated, indicating that the MSFP-based memristors have shown good device-to-device stability (Supplementary Figure 6c).

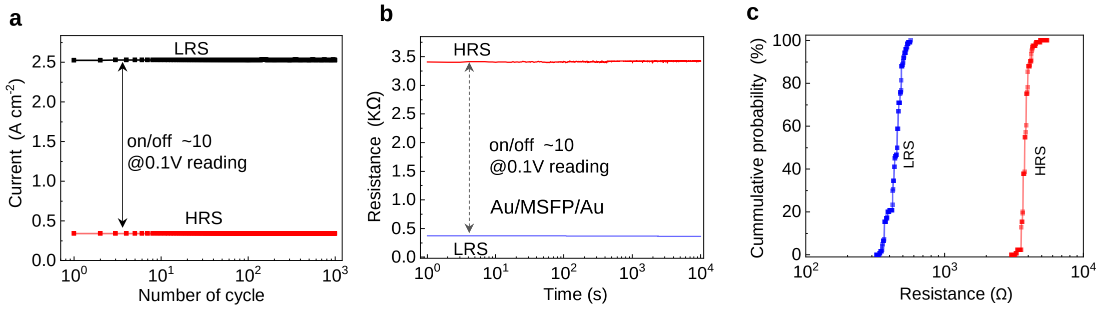

Supplementary Fig. 6. Endurance, retention, and device-to-device stability of the electrical resistive switching in the MSFP-based memory device. a. Endurance test for 1000 cycles. **b**. Retention for the LRS and HRS. **c**, Cumulative plot of the HRS and LRS in the 100 MSPF memory devices.

When a single electrical pulse with a voltage amplitude of 0.7 V and pulse width of 50μs is applied to the device, the device conductance quickly changes from a lower conductance state to a higher conductance state. The response time can be extracted as ~10μs, as shown in Supplementary Figure 7.

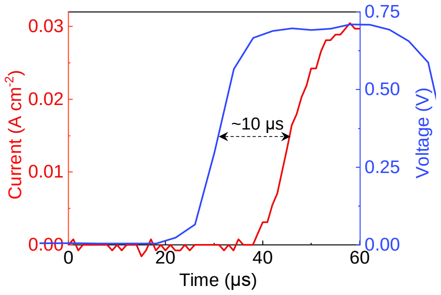

Supplementary Fig. 7. Conductance response in the Au/MSFP/Au resistive memory. Current response under the electrical pulse stimuli with amplitude of 0.7 V and pulse width of 50 μs, indicating a response time of ~10μs for the MSFP-based resistive memory.

Supplementary Figures 8a-c are the typical *I-V* curves of the SFP memory with the precursor dialyzing time from 72 to 36 hours, respectively, all demonstrating digital-

type switching featured by abrupt SET and RESET process, which may be attributed to the formation of the Li^+-based conduction paths.

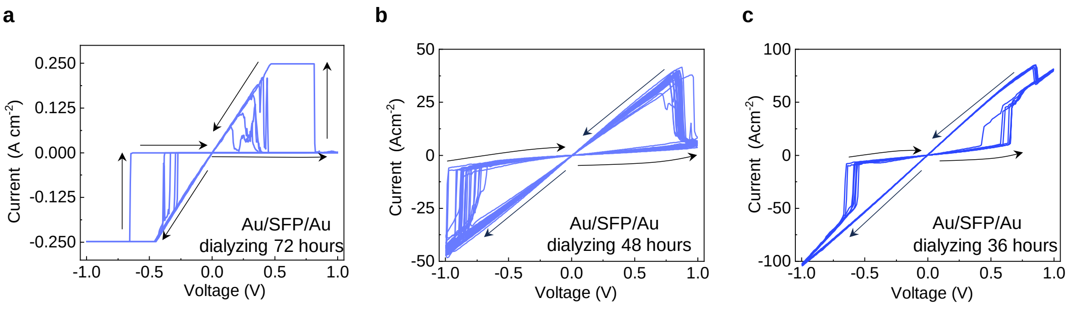

Supplementary Fig. 8. Memory switching behaviours of the SFP device with different dialyzing times from 72 to 36 hours.

The multilevel storage of 16 conductance states and retention properties of the MSFP memory device is demonstrated in Supplementary Figure 9. The 16 conductance states are programmed by consecutive electrical pulse groups, with each group composed of five pairs of a programming pulse (0.7V, 50μs) followed by a reading pulse (0.1V, 50μs). All 16 conductance states exhibit nonvolatile properties with a retention time of up to 10^3 seconds, as shown in Supplementary Figure 9.

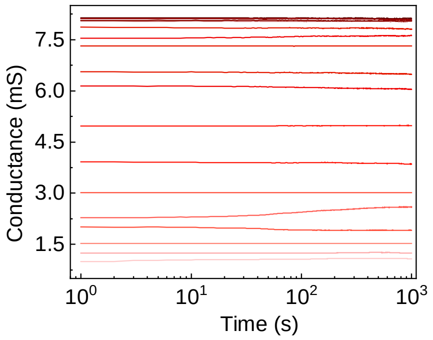

Supplementary Fig. 9. The 16 nonvolatile conductance states under the 16 electrical pulse groups that each group is composed of ten constant pulses (0.7V, 50μs) followed by reading voltage 0.1V for 1000 seconds.

Supplementary Figure 10a (LTP in Figure 2d) exhibits the conductance potentiation

with consecutively applied 50 positive pulses (0.7V, 50μs) for the long-term potentiation (LTP) process and the conductance retention after the removal of the LTP stimulus. It can be noted the conductance state can be well maintained. Supplementary Figure 10b (LTD in Figure 2d) presents the LTD process with the consecutively applied 50 negative pulses (-0.7V, 50μs). The programmed conductance state of LTD can also be maintained after removing the LTD stimulus. The read voltage for LTP and LTD and retention tests are controlled as 0.1 V. Therefore, the conductance states programmed by the LTP and LTP pulses demonstrate good nonvolatility.

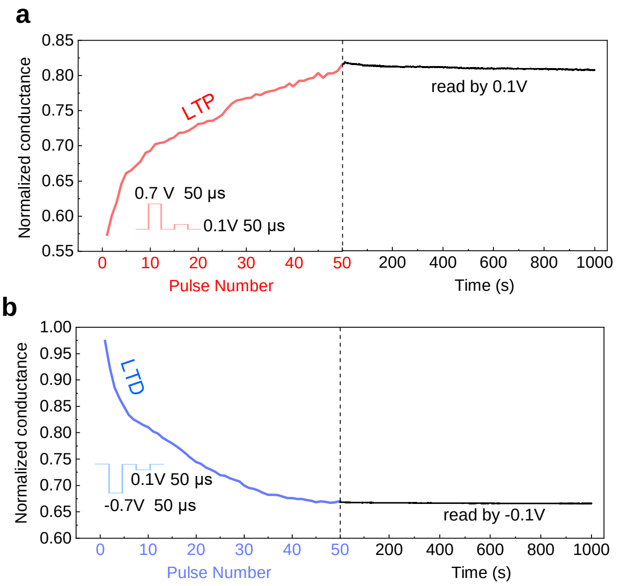

Supplementary Fig. 10. LTP and LTD and the retention properties of the Au/MSFP/Au resistive memory. **a**. The conductance programmed by the 50 positive pulses (0.7V, 50μs) for LTP can be well maintained after removing the pulse stimulus. **b**. The conductance programmed by 50 negative pulses (-0.7V, 50μs) for LTD can also be well maintained after removing the pulse stimulus.

Supplementary Note 3

The physical mechanism of the analogue resistive switching behaviours. The defect-related Poisson equation is described as follows [9, 10]:

$$\frac{\varepsilon_0 \varepsilon_r}{e} \frac{dE(x)}{d(x)} = n(x) \quad (1)$$

Where the $n(x)$, $E(x)$ represent the charge density and electric field respectively. The ε_0, ε_r, and e are the vacuum permittivity, relative permittivity and electron, respectively. The charge injection is controlled by the external electric field. At a low bias voltage, most of the injected electrons are captured in the defect sites. In this case, the concentration of the free electrons (n_f) is negligible. The N_t and n_t are defined as the total defects and the electron-filled defects. The n_t is given by:

$$n_t = \frac{N_t}{1+exp^{(\frac{E_t - E_F}{kT})}} \quad (2)$$

Thus, the charge density can be represented as $n = n_f + n_t$. In this case, the Poisson equation can be re-written by:

$$\frac{\varepsilon_0 \varepsilon_r}{e} \frac{dE(x)}{d(x)} = (n_f + n_t)\ (x) \quad (3)$$

Therefore, the current density can be described by the Mott-Gurrey law[11, 12]:

$$J = \frac{9}{8} \varepsilon_0 \varepsilon_r \theta \mu \frac{V^2}{L^2} \quad (4)$$

where $\theta = n_f/(n_f + n_t)$ is the proportion of free electrons. The μ and L denote the mobility and thickness of the switching function layer, respectively. Therefore, the charge density is in the state of $n_f << n_t$ under the low bias voltage. With a relatively high voltage, the conduction is dominated by the Mott-Gurrey law; in other words, the current-voltage relation obeys the $I \sim V^{m=2}$. The current-voltage relation follows the I~Vm>2 in the large bias voltage region. The double logarithm *I-V* fitting is conducted using the continuous I-V curves (Supplementary Figure 11a). The transition from the low bias voltage region to a relatively high bias voltage region occurs at 0.025 V. The transition from the relatively high bias voltage region to the high bias voltage region occurs at different voltages (0.67, 0.61, 0.57, 0.54 V) for the 1st, 5th, 10th and 15th cycles,

respectively. Therefore, the defect concentration in the MSFP switching layer can be calculated by [10]:

$$N_t = \frac{2\varepsilon_0\varepsilon_r}{eL^2}V_t \qquad (5)$$

Where the V_t is the transition voltage. According to equation 5, the defect concentrations can be calculated at different bias voltages, respectively (Supplementary Figure 11b). The defect concentration is 0.11×10^{16} for the low bias voltage region, decreasing from 0.27×1016 to 0.14×1016 with continuous *I-V* sweepings. Therefore, the electrons are iteratively stored in the MSFP function layer.

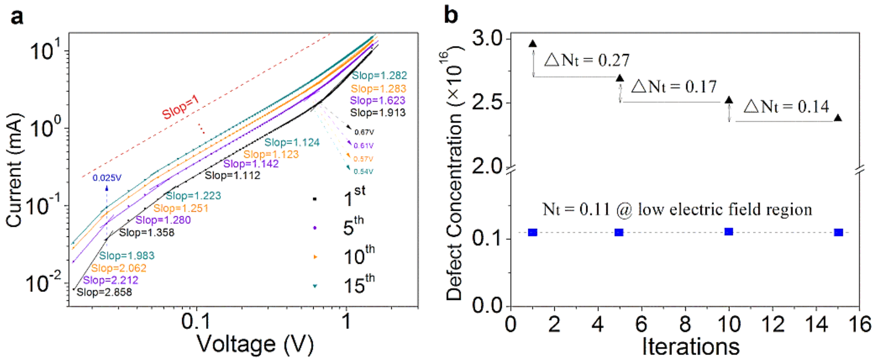

Supplementary Fig. 11. Calculation of the defect concentrations in the MSFP-based memory device. a. The space charge limited current (SCLC) fitting for different bias voltage regions. **b**. Calculation of the concentration of defects under different *I-V* sweeping cycles (iterations).

Supplementary Figures 12a-c show the *I-V* resistive switching curves of the MSFP-based resistive memories with different Li^+ concentrations and the corresponding trap concentrations obtained by controlling the precursor dialyzing times. The MSFP resistive memory devices with dialyzing times of 36 and 48 hours exhibit continuous and graded switching behaviours, while the MSFP memory with dialyzing times of 72 hours exhibits abrupt switching. For the MSFP memory device with a dialyzing time of 72 hours, the device has the least Li^+ and trapping sites, which cannot provide the sites

for continuous electron trapping. In comparison, the devices with dialyzing times of 36 hours and 48 hours have more trapping sites than those of 72 hours, therefore exhibiting continuous and graded switching.

Comparing the switching between the devices with the dialyzing times of 36 hours and 48 hours, the device with the dialyzing time of 36 hours shows higher current density and a relatively unstable switching effect, while the device with the precursor dialyzing time of 48 hours shows lower current density and better analogue switching behaviours. This may be attributed to that a longer dialyzing time induces less Li^+ and fewer trap states inside the MSFP thin films. Because of the lower trap concentration, the MSFP memory with a 48-hour precursor dialyzing time shows a lower current density at HRS and more stable switching than that with a 36-hour precursor dialyzing time. Although a relatively high Li^+ concentration and trap concentration will lead to a higher on/off ratio in the MSFP device with a dialyzing time of 36 hours, the device shows a higher HRS current density and relatively unstable switching.

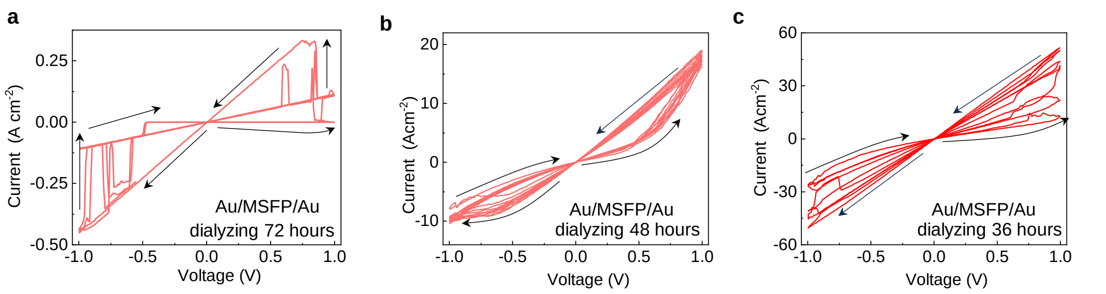

Supplementary Fig. 12. Switching memory behaviours of the MSFP device with different dialyzing times from 72 to 36 hours.

Since the residual ion, such as $Li^{+,}$ can introduce a certain number of trap sites in the MSFP function layers [1-3], the MSFP memory devices with different Li^+

concentrations and, thus, different trapping concentrations are fabricated to prove the switching mechanism further. The Li^+ concentrations can be modulated through the precursor dialyzing times. Therefore, we prepared the MSFP memory devices with different precursor dialyzing times of 36, 48 and 72 hours, respectively, as shown in Supplementary Table 1 and estimated the number of Li^+ in the MSFP materials by the inductively coupled plasma (ICP) with mass spectrometry, as shown in Supplementary Table 1. The results suggest that the MSFP memory device with a dialyzing time of 72 hours with the least number of Li^+ and trapping sites shows abrupt switching instead of continuous switching.

In comparison, the devices with dialyzing times of 36 hours and 48 hours and the correspondingly more trapping sites all exhibit continuous and graded switching. The device with the 36-hour dialyzing time shows a higher on/off ratio than the 48-hour dialyzing time because of the higher trap concentration, which also agrees with the proposed switching mechanism. However, the higher trap concentration could also lead to a relatively unstable switching and higher HRS current density.

In summary, the analogue switching behaviours mainly rely on the electron soft filling in the Li^+-introduced traps in the MSFP thin film. The device shows a higher on/off ratio when the trap concentration increases. However, the higher trap concentration could also lead to a relatively unstable switching and higher HRS current density. Therefore, the MSFP memory with dialyzing 48 hours was used in this work.

Supplementary table 1. Li-ion concentration in the MSFP material measured by inductively coupled plasma (ICP) with mass spectrometry.

Dialysis time (hours)	Li-ion concentration (mg/kg)
36	65.30
48	56.08
72	48.74

Supplementary Note 4

Optical resistive switching. The device-to-device and cycle-to-cycle repeatabilities in the ORRAM mode device are shown in Supplementary Figure 13. The programmed resistance states at the intermediate state, PPM state and NPM state are recorded by a small voltage sweep from 0-0.2 V, which will not cause the resistance change for 100 cycles, indicating small cycle-to-cycle variation (Supplementary Figure 13a). Supplementary Figure 13b shows the cumulative plot of the intermediate, PPM, and NPM states in 100 MSFP memory devices, suggesting good device-to-device reproducibility for the ORRAM mode.

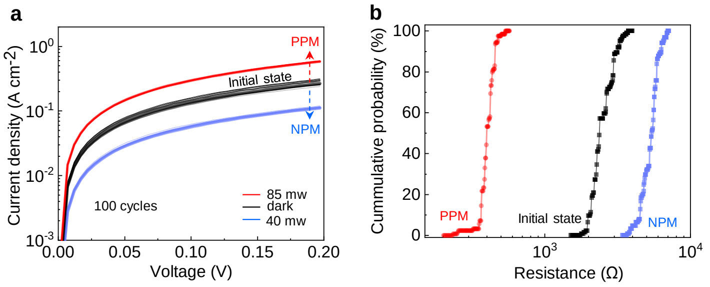

Supplementary Fig. 13. Device-to-device and cycle-to-cycle stabilities in the MSFP memory device with ORRAM mode operation. a. The cyclically programmed resistance states of the intermediate, PPM, and NPM states. **b**. The cumulative plot of the intermediate, PPM, and NPM states in 100 different MSFP memory devices.

The protein secondary structures, including the side chain (*s-c*), random coil (*r-c*), alpha helix (*a-h*), beta-sheet (*β-s*), and beta-turn (*β-t*), were quantized in the first amide region (1470~1720 cm^{-1}). For the SFP film, the quantification results in the amide first region illustrate that the mole ratios of *s-c*, *β-s*, *r-c*, *a-h*, and *β-t* secondary structures are 4.88%, 35.81%, 24.30%, 18.03%, and 16.98%, respectively, as shown in Supplementary Figure 14a. For the MSFP thin film, the secondary structures are changed. The mole ratios of s-c, β-s, r-c, a-h, and β-t secondary structures in the MSFP film become 8.39%, 53.09%, 14.60%, 10.75% and 13.17%, respectively. The comparisons of the secondary structures in SFP and MSFP are listed in Supplementary Table 2. Notably, the secondary structures in the MSFP undergo alteration under 405 nm light illumination, which is responsible for the optically induced resistance change. Under the 60 mW 405 nm light illumination, the mole ratios of *s-c, β-s, r-c, a-h,* and *β-t* secondary structures in the MSFP film change to 6.59%, 54.51%, 13.04%, 8.85%, and 17.01%, respectively (Supplementary Figure 14b). The secondary structure changes under the light intensities varying from 40 to 80 mW were listed in Supplementary Table 3, suggesting that the negative photoconductance memory effect (NPM) and the positive photoconductance effect (PPM) originated from the change of the mole ratio of these secondary structures. The secondary structures with layer-by-layer structures, such as the β-s secondary structures, contribute to higher conductivity while the conductivity change of turn structure or other conformation is reversed. Therefore, the PPM effect can be mainly ascribed to the net increase of the mole ratio of *β-s*, while the

NMP effect can be attributed primarily to the net increase of the mole ratio of *β-t* and other secondary structures.

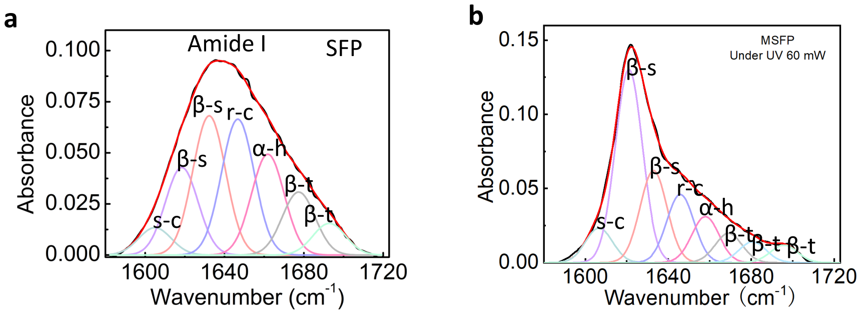

Supplementary Fig. 14. Secondary structure characterizations of the SFP and MSFP thin films. a. The FTIR spectra of the SFP thin film and the secondary structure dependent fitting in the amide first region. **b**. The secondary structure of the MSFP thin film under 60mW UV light.

Supplementary Table 2. Comparison of the mole ratios of secondary structures in SFP and MSFP thin films.

	s-c	*β-s*	*r-c*	*a-h*	*β-t*
SFP	4.88%	35.81%	24.30%	18.03%	16.98%
MSFP	8.39%	53.09%	14.60%	10.75%	13.17%

Supplementary Table 3. Secondary structures of MSFP under different UV light intensities.

	s-c	*β-s*	*r-c*	*a-h*	*β-t*
40mW	6.89%	55.76%	13.52%	9.32%	14.51%
60mW	6.59%	54.51%	13.04%	8.85%	17.01%
80mW	6.96%	59.30%	13.16%	8.17%	12.41%

First, for the effects of the MSFP thickness on the optical switching behaviours, we studied the optical switching behaviours in the MSFP memory with different MSFP thicknesses of ~63, ~97, and ~186nm, respectively, as shown in Supplementary Figure 15. The MSFP's thickness greatly impacts the resistive switching behaviours, including current density, resistance ratio, and stability. The memory device with a 63 nm MSFP

function layer shows a relatively high current density of 8~25 A cm^{-2} at 0.2V and a small resistance ratio (~3) between PPM and NPM states (Supplementary Figure 15a). A higher resistance ratio (~10) and lower current density (0.35~5 A cm^{-2} at 0.2V) are obtained in the MSFP memory with ~97 nm MSFP thin film (Supplementary Figure 15b), which may be attributed to the higher secondary structure change volume compared with that of 63 nm. However, the higher resistance states and even smaller resistance ratio (~2) are observed in the MSFP memory with an MSFP thickness of 186 nm, which may be attributed to the fact that the conversion volume of secondary structure in the MSFP thin film is limited under the light illumination with a specific power, therefore the secondary structure conversion ratio and the resistance change ratio is relatively small when the MSFP thin film thickness is relatively thick (186 nm), and the as-prepared MSFP memory device is already at very HRS (Supplementary Figure 15c).

For the area influences on the conductance, the optical switching behaviours of MSFP memory devices with different device areas of 100×100μm^2, 150×150μm^2 and 200×200 μm^2 are studied as shown in Supplementary Figure 15d. It can be noted that the memory device with a large area exhibits a wider conductance range for both the NPM and PPM effects. The memory cell with an area of 200×200μm^2 shows an optical switching ratio of ~10, while this ratio respectively decreases to ~8 and ~5 when the area decreases to 150×150μm^2 and 100×100μm^2, as shown in Supplementary Figure 15d. The photoconductance change ratio shows increasing trends with the increased device area since the large area corresponds to a higher volume of the secondary

structure change, thus a larger photoconductance change. The area-dependent switching is also consistent with the conductance change in the MSFP thin films that arises from the secondary structure change.

When changing the device area and the MSFP thin film thickness, the critical intensity is nearly unchanged. The crucial intensity in the change from PPM to NPM effect mainly depends on the heat provided by the light [13-15]. Therefore, the threshold heat and optical power for turning PPM to NPM are nearly identical for devices with different areas or thicknesses.

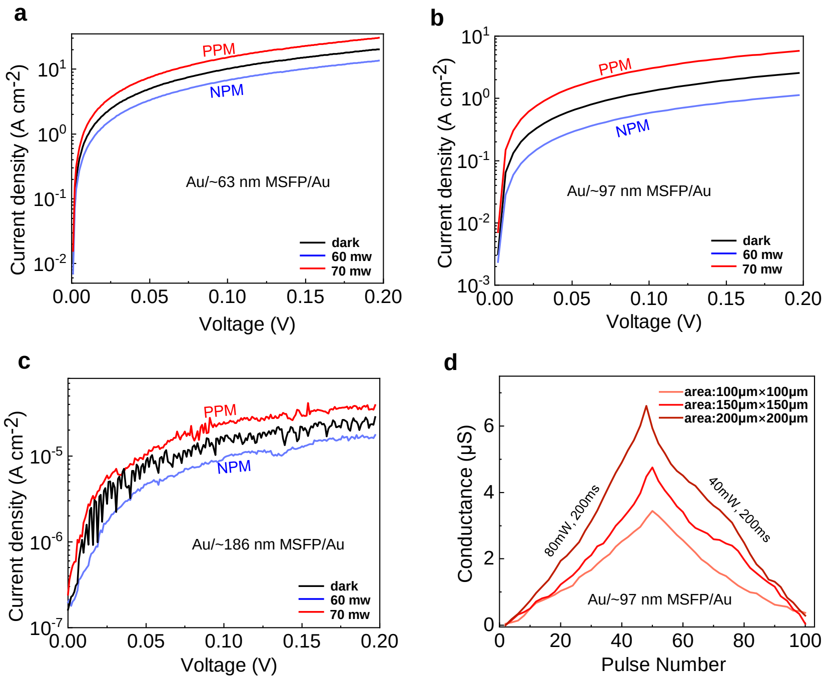

Supplementary Fig. 15. The influences of device area and MSFP thickness on the optical switching behaviours. a-c, The optical switching behaviours in the MSFP memory devices with different MSFP thicknesses of ~63, ~97, and ~186nm, respectively. **d,** Device area dependent optical LTPs and LTDs in the memory cell with different device areas of 100×100, 150×150 and 200×200μm^2.

To investigate the impact of the electrical switching on the optical switching, the Au/MSFP/Au resistive memory is first set to the low resistance state (LRS) by 100

electrical pulses (0.7V, 50μs). Then, at this LRS triggered by the electrical pulses, we further employ 100 PPM optical pulses (80mW, 200ms) to set the device to a lower LRS, and then this lower LRS can be reset back to the low resistance state (LRS) through 100 NPM optical pulses (40mW, 200ms). The LRS is reset to its initial HRS baseline through 100 electrical pulses (-0.7V, 50μs). After the above operation, the memory device can still be set and reset by the light pulses (set by 50 optical pulses (80mW, 200ms) and then reset by 50 optical pulses (40mW, 200ms)), showing unaffected optical switching behaviours (Supplementary Figure 16). Therefore, the electrical switching nearly does not impact the optical switching behaviours.

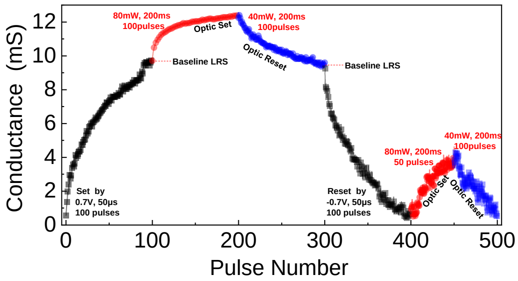

Supplementary Fig. 16. The conductance changes with the first 100 electrical pulses (0.7V, 50μs) and 100 optical pulses (80mW, 200ms) for the set process and 100 optical pulses (40mW, 200ms) and 100 electrical pulses (-0.7V, 50μs) for the reset process, followed with another 50 optical pulses (80mW, 200ms) for optical set and 50 optical pulses (40mW, 200ms) for optical reset.

Supplementary Table 4. Comparison of the optoelectronic memory devices based on different photoelectronic function materials.

Device	Material	Optical switching memory	Fully optical operation	Optical response	Optical retention	Electrical switching memory	Electrical switching type	Electrical retention	Electrical operation voltage	Energy Consu mption	On/off ratio	Multimodal	Applications	Full hardware system
Memristor (Ref.16)	MoO_x	Yes, opt. memory	No	~100ms	>10^2s	No	No	-	2.5V/0.1V	-	-	No	contrast enhancement; denoising	No
Memristor + diode (Ref.17)	InGaAs-based	No, opt. response	No	~100ms	volatile	Yes	digital	>10^4s	~5V/~1V	-	~10^2	Yes	image classification	No
Memristor (Ref. 18)	$GeSe_3$	Yes, opt.	No	~5s	~10^2s	Yes	digital	>10^4s	~0.6/-	0.39mJ	~10^3	No	logical operation	No
Memristor (Ref. 19)	Graphene	No, opt.	No	~2s	volatile	Yes	digital	>10^4s	~6V/0V	-	~10^3	No	image classification	No
Memristor (Ref. 20)	InGaZnO	Yes, opt. memory	Yes	~2s	~10^4s	Yes	analogue	>10^4s	~2.0V/10mV	-	~5	No	-	No
Transistor (Refs. 21-24)	WSe_2-based	Yes, opt. memory	No	~200ms	~10^2s	-	No	-	1.5~9V/-	-	-	No	motion detection;	No
Transistor (Refs.25-31)	MoS_2-based	No, opt. response	No	~5ms	volatile	-	-No	-	3~6V/-	-	-	No	perception encoding;	No
Transistor (Refs. 32-37)	Organic-inorganic-based	No, opt. response	No	~5ms	volatile	-	-No	-	~40V/	-	-	No	-	No
Memristor (Our work)	modified silk fibroin protein	Yes opt. memory	Yes	~100ms	>10^4s	Yes	analogue	>10^4s	1V/0.1V	~250pJ	~10	Yes	feature extraction; contrast enhancement; background denoising; image recognition	Yes

Supplementary Note 5

Demonstration of the image preprocessing based on the ORRAM array. The 12×12 input image is mapped on the 12×12 MSFP memory array according to 12 different light intensities (12 grey scales) at a constant wavelength of 405nm and a continuous pulse number of 50, as shown in Supplementary Figure 17. The mapping detail is as follows:

As shown in Figure Supplementary Figure 17, the image mapping onto a 12×12 device array is conducted by applying the light pulses (pulse number of 50, pulse width of 100 ms, and wavelength of 405nm) to each device in the device array according to pixel intensities in the input image. Specifically, in the mapping process, each pixel corresponds to each device. The input images are grayscale. Each image has 12 grey scales, corresponding to 12 different light intensities of 40, 45, 50, 55, 60, 70, 75, 80, 85, 90, 95, and 100 mW. Each input image is composed of two parts: body pattern pixels and background pixels, in which the body pattern pixels with relatively higher brightness corresponds to the higher light intensities of 70, 75, 80, 85, 90, 95, and 100 mW, while the background pixels with lower brightness corresponds to the lower light intensities of 40, 45, 50, 55, and 60mW.

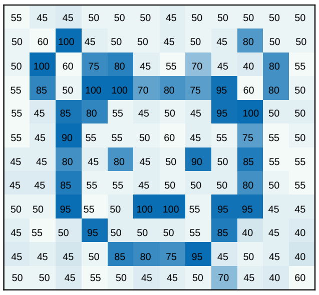

Supplementary Fig. 17. The image mapping on the ORRAM mode array according to different light intensities (12 grey scales) at a constant wavelength of 405nm and a constant pulse number of 50.

The image contrast enhancement is dependent on the epochs. Supplementary Figure 18 shows the enhanced image contrast versus pulse number and the output current density through the PPM effect (corresponding to the body pixels with higher light intensities) and NPM effect (corresponding to the background pixels with lower light intensities) versus pulse number. It suggests that the contrast between the body pixels and background pixels is gradually enhanced as the pulse number increases, which is due to the enlarged output current between the body pixels and background pixels through PPM and NPM effects with the increased pulse number as shown in Supplementary Figure 18. As the pulse number increases to 25, the image contrast can be improved six times compared with the original image.

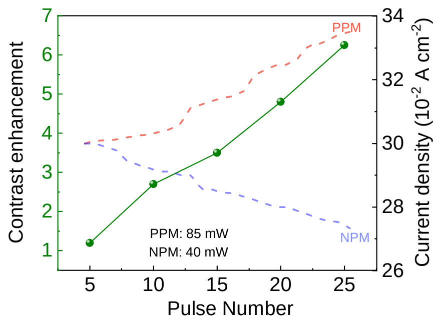

Supplementary Fig. 18. Image contrast enhancement as a function of pulse number (epochs).

Supplementary Note 6

Neural network structure and application demonstration. A complete CNN neural network consists of one layer of ORRAM convolution, max pooling, ReLU activation function, one ERRAM convolution, max pooling, ReLU activation function, and one fully connected layer, as shown in Supplementary Figure 19. For the full hardware implementation, the ORRAM array first perceives image information and then executes

the in-sensor convolution operation for image feature extraction. The values of the convolution kernels, ranging from -1 to 1, are mapped to a voltage range of -0.2V to 0.2V generated by the DAC and then input into the ORRAM array. The TIA on the column acts as a virtual ground, converting the incoming current into voltage, which is further collected and converted into a digital signal by the ADC. Based on these extracted features, a 2×2 max-pooling operation and ReLU activation function are performed within the ARM core. Subsequently, these features are further input into ERRAM for convolution and fully connected layer computations. Due to the ReLU activation function, all feature values become positive. When the features are input into the ERRAM array, all feature values are normalized to 0 to 1 and then mapped to the voltage range of 0 to 0.2V. These normalized feature values are converted into voltage pulses using the DAC and are input into the ERRAM. The convolution operation is performed first, with the TIAs on the columns acting as virtual grounds, collecting the currents on the columns and converting them back into voltages. Afterwards, a 2×2 max-pooling operation and ReLU activation function are executed within the ARM core. Finally, the processed feature values are input into the ERRAM "fully connected layer" to perform classification and output the results.

First, the image is input into a 12×12 ORRAM layer, and the convolution is in-sensor performed with a 1×3×3×3 (depth × weight × weight × batch) kernel, resulting in a 12×12×3 feature map. After 2×2 max pooling and ReLU activation operations are performed within the ARM core, the feature map is reduced to 6×6×3. These features are then input into the ERRAM layer for convolution operation with a 3×3×3×1 kernel,

resulting in a feature size of 6×6×1. Similar to the previous 2×2 max pooling and ReLU activation operations, the 6×6×1 feature map is reduced to 3×3×1. The nine features are then input into a 9×3 fully connected layer and classified.

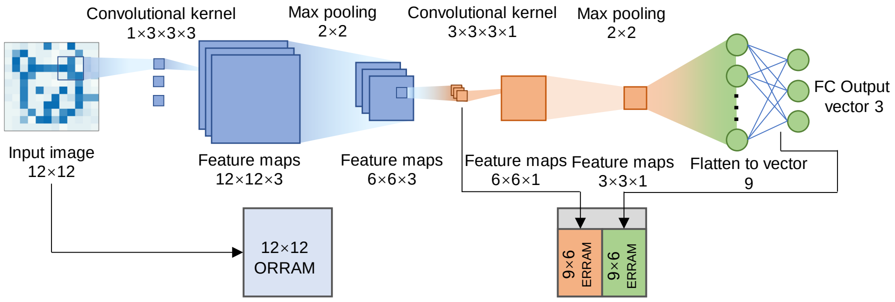

Supplementary Fig. 19. The whole CNN neural network structure implemented based on the ORRAM mode array and ERRAM mode array. The image features are first extracted through the convolution operation in the ORRAM array, followed by pooling and activation functions, resulting in a feature size 6×6×3. These features are then processed through the convolution operation in the ERRAM array, followed by pooling and activation functions, resulting in a feature size of 3×3×1. These features are input into a 9×3 fully connected layer for image classification.

We used MNIST handwritten digit image for image preprocessing simulation according to the ORRAM mode device characteristics (Supplementary Figure 20). Based on the device-level NPM and PPM characteristics, image denoising and contrast enhancement were realized through the ORRAMs. Additionally, based on the in-sensor convolution capability in the ORRAM mode array, the edge feature in the images can be effectively extracted.

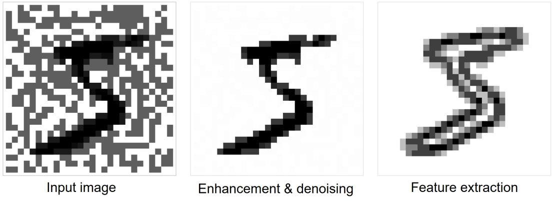

Supplementary Fig. 20. Simulations of the preprocessing capabilities of the ORRAM mode array for larger-size images.

Supplementary Figure 21a-c shows the conductance programming capabilities in the 12×12 MSFP ERRAM mode array. A 12×12 ERRAM mode crossbar array is first randomly programmed, resulting in a random conductance distribution (State 1). State 2 shows the randomly programmed conductance states in the ERRAM mode array using the pulses with input voltage ranging from 0.7V to 0.7V. State 3 shows the conductance states in the ERRAM mode array with columns 5, 9, and 10 selected for programming (Supplementary Figures 21a-c). This demonstrates ERRAM's programmability for high-level image processing. Supplementary Figure 21d shows the processing of “MNIST handwritten digit” images through the convolution operations implemented by the ERRAM mode array-based hardware, such as image blurring.

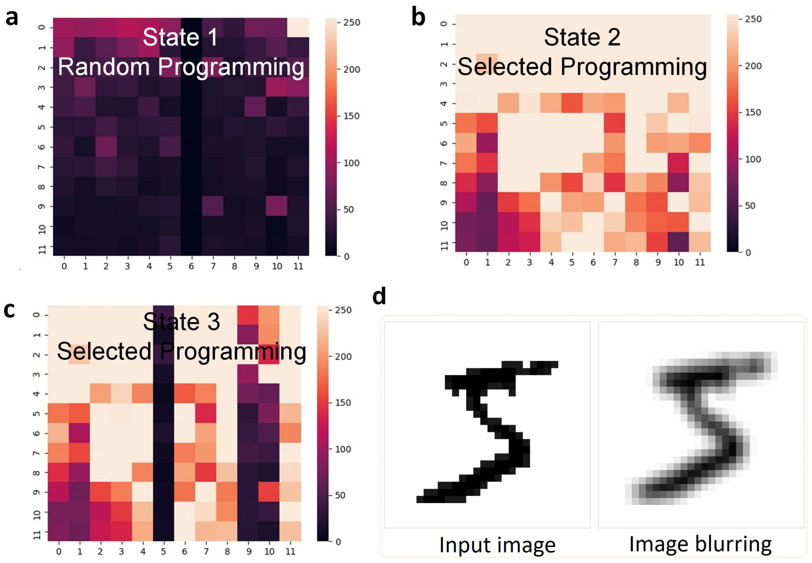

Supplementary Fig. 21. a-c. Conductance programming in the MSFP-based ERRAM mode array. **d**. MNIST handwritten digit image processing through the convolution operations implemented by the ERRAM mode array-based hardware.

Supplementary Note 7

System performance (The calculation is estimated according to the peripheral circuits with 28 nm technology node).

Supplementary Table 5: The system performance based on the ORRAM mode and ERRAM mode array when our memory device is scaled down to 0.5μm×0.5μm.

Performance	12×12×2×2/25ns=23.04GOPS
Power	3.8pJ/25ns=0.152mW
Area	0.001237mm^2
Energy efficiency	23.04GOPS/0.152mW=151.579TOPS/W
Performance density	23.04GOPS/0.001237mm^2=18.626TOPS/mm^2

Supplementary Table 6: System performance comparison.

	Ref. 38	Ref. 39	Ref. 40	This work
Memory	Transistor +RRAM	Transistor +RRAM	Transistor +RRAM	ORRAM+ERRAM
Energy efficiency (TOPS/W)	-	75.17	11.014	151.579
Performance density (TOPS/mm^2)	8.5	7.008	1.164	18.626
Area(mm^2)	0.217	0.0263	0.0704	0.001237
Additional CMOS image sensor required	Yes	Yes	Yes	No
In-sensor computing	NO	NO	NO	Yes

References

1. Wang, H. et al. Configurable resistive switching between memory and threshold characteristics for protein-based devices. *Adv. Funct. Mater.* **25**, 3825-3831 (2015).
2. Wang, H. et al. Physically transient resistive switching memory based on silk protein. *Small* **12**, 2715-2719 (2016).
3. Jung, H. L. et al. Understanding hydroscopic properties of silk fibroin and its use as a gate-dielectric in organic field-effect transistors, *Org. Electron.* **59**, 213-219 (2018).
4. Reizabal, A. et al. Optically transparent silk fibroin/silver nanowire composites for piezoresistive sensing and object recognitions, *J. Mater. Chem. C*. **8**, 13053-13062 (2020).
5. Zhou, G. et al. Artificial and wearable albumen protein memristor arrays with integrated memory logic gate functionality. *Mater. Horizons*. **6**, 1877-1882 (2019).
6. Wang, W., et al. An analogue memristor made of silk fibroin polymer. *J. Mater. Chem. C.* **9**, 14583-14588 (2021).
7. Wang, W., et al. Moisture influence in emerging neuromorphic device. *Front. Phys.* **18**, 53601 (2023).
8. Zhou, G., et al. PbI_3^- ion abnormal migration in $CH_3NH_3PbI_xCl_{3-x}$ ultralong single nanowire for resistive switching memories. *Mater. Characterization.* **199**, 112762 (2023).
9. Yang, X., et al. Investigation of a submerging redox behavior in Fe_2O_3 solid electrolyte for resistive switching memory. *Appl. Phys. Lett.* **114**, 163506 (2019).
10. Carbone, A., et al. Space-charge-limited current fluctuations in organic semiconductors. *Phys. Rev. Lett.* **95**, 236601 (2005).
11. Zhang, P., et al. 100 years of the physics of diodes. *Appl. Phys. Rev*. **4**, 011304 (2017).
12. Zhou, G., et al. Investigation of the behaviour of electronic resistive switching memory based on $MoSe_2$-doped ultralong Se microwires. *Appl. Phys. Lett.* **109**, 143904 (2016).
13. Wang, W., et al. An analogue memristor made of silk fibroin polymer. *J. Mater. Chem. C.* **9**, 14583-14588 (2021).
14. Min, K., Umar, M., Ryu, S., Lee, S., Kim, S. Silk protein as a new optically transparent adhesion layer for an ultra-smooth sub-10 nm gold layer. *Nanotechnology* **28**, 115201 (2017).
15. Shi, C., et al. New silk road: from mesoscopic reconstruction/functionalization to flexible meso-electronics/photonics based on cocoon silk materials. *Adv. Mater.* **33**, 2005910 (2021).
16. Zhou, F., et al. Optoelectronic resistive random-access memory for neuromorphic vision sensors. *Nat. Nanotechnol.* **14**, 776-782 (2019).
17. Lee, D., et al. In-sensor image memorization and encoding via optical neurons for bio-stimulus domain reduction towards visual cognitive processing. *Nat. Commun*. **13**, 5223 (2022).

18. Syed, S., et al. Chalcogenide optomemristors for multi-factor neuromorphic computation. *Nat. Commun.* **13**, 2247 (2022).
19. Fu, X., et al. Graphene/$MoS_{2-x}O_x$/graphene photomemristor with tunable non-volatile responsivities for neuromorphic vision processing. *Light. Sci. Appl.* **12**, 39 (2023).
20. Hu, L., et al. All-optically controlled memristor for optoelectronic neuromorphic computing, *Adv. Funct. Mater.* **31**, 2005582 (2021).
21. Mennel, L., et al. Ultrafast machine vision with 2D material neural network image sensors. *Nature* **579**, 62-66 (2020).
22. Chen, H., Xue, X., Liu, C., Fang, J., Wang, Z., Wang, J., & Zhou, P. Logic gates based on neuristors made from two-dimensional materials. *Nat. Electron.* **4**, 399-404 (2021).
23. Wang, C. Y. et al. Gate-tunable van der Waals heterostructure for reconfigurable neural network vision sensor. *Sci. Adv.* **6**, eaba6173 (2020).
24. Zhang, Z., et al. All-in-one two-dimensional retinomorphic hardware device for motion detection and recognition, *Nat. Nanotechnol.* **17**, 27-32 (2022).
25. Chen, J., Chai, Y., et al. Optoelectronic graded neurons for bioinspired in-sensor motion perception. *Nat. Nanotechnol.* **18**, 882-888 (2023)
26. Liao, F., Chai, Y., et al. Bioinspired in-sensor visual adaptation for accurate perception. *Nat. Electron*. **5**, 84-91 (2022).
27. Yu, J., et al. Bioinspired mechano-photonic artificial synapse based on graphene /MoS_2 heterostructure. *Sci. Adv.* **7**, eabd9117 (2021).
28. Syed, S., et al. Atomically thin optomemristive feedback neurons. *Nat. Nanotechnol.* **18**, 1036-1043 (2023).
29. Wang, F., et al. A two-dimensional mid-infrared optoelectronic retina enabling simultaneous perception and encoding. *Nat. Commun.* **14**, 1938 (2023).
30. Dodda, A., et al. Active pixel sensor matrix based on monolayer MoS_2 phototransistor array. *Nat. Mater.* **21**, 1379-1387 (2022).
31. Ma, S., et al. A 619-pixel machine vision enhancement chip based on two-dimensional semiconductors. *Sci. Adv.* **8**, eabn9328 (2022).
32. Chen, K., et al. Organic optoelectronic synapse based on photon-modulated electrochemical doping. *Nat. Photon.* **17**, 629-637 (2023).
33. Zhu, Q., et al. A flexible ultrasensitive optoelectronic sensor array for neuromorphic vision system. *Nat. Commun.* **12**, 1798 (2021).
34. Jiang, T., et al. Tetrachromatic vision-inspired neuromorphic sensors with ultraweak ultraviolet detection. *Nat. Commun.* **14**, 2281 (2023).

35. Lee, S., et al. Programmable black phosphorus image sensor for broadband optoelectronic edge computing. *Nat. Commun.* **13**,1485 (2022).
36. Xu, Y., et al. Optically readable organic electrochemical synaptic transistors for neuromorphic photonic image processing. *Nano Lett.* **23**, 5264-5271 (2023).
37. He, S., et al. Reconfigurable optoelectronic synaptic transistor with stable Zr-$CsPbI_3$ nanocrystals for visuomorphic computing, *Adv. Mater.* **35**, 2208479 (2023).
38. Correll J. M., Jie L., Song S., et al. An 8-bit 20.7 TOPS/W multi-level cell ReRAM-based compute engine. *VLSI Technology and Circuits*. 264-265 (2022).
39. Spetalnick S. D., Chang M., Konno S., et al. A 2.38M cells/mm^2 9.81-350 TOPS/W RRAM compute-in-memory macro in 40nm CMOS with hybrid offset/I OFF cancellation and I cell R BLSL drop mitigation. *VLSI Technology and Circuits*. 1-2 (2023)
40. Yao P., Wu H., Gao B., et al. Fully hardware-implemented memristor convolutional neural network. *Nature* **577**, 641-646 (2020).
